# Wound infection caused by Photobacterium damselae in a 32-year-old woman: case report and review of the literature

**DOI:** 10.3205/id000067

**Published:** 2020-11-17

**Authors:** Percy Schröttner, Eric Tille, Christian Lück, Boyke Bunk

**Affiliations:** 1 Institut für Medizinische Mikrobiologie und Hygiene, Medizinische Fakultät Carl Gustav Carus, Technische Universität Dresden, Germany; 2 UniversitätsCentrum für Orthopädie, Unfall- und Plastische Chirurgie (OUPC), Universitätsklinikum Dresden, Medizinische Fakultät Carl Gustav Carus Dresden, Germany; 3 Leibniz-Institut DSMZ – Deutsche Sammlung für Mikroorganismen und Zellkulturen GmbH, Braunschweig, Germany

**Keywords:** Photobacterium damselae, wound infection, whole genome data, MALDI TOF MS, antimicrobial profile

## Abstract

The case of a 32-year-old woman is reported, who was affected by a persisting wound infection caused by *Photobacterium damselae* after an accident in the Mediterranean Sea. Besides the clinical case, microbiological characteristics based on the phenotypic and genotypic description of the isolate (including whole genome data) are presented and discussed.

## Zusammenfassung

Wir berichten über den Fall einer 32-jährigen Frau, die nach einem Badeunfall im Mittelmeer von einer persistierenden Wundinfektion, welche durch *Photobacterium* *damselae* verursacht wurde, betroffen war. Neben der Beschreibung des klinischen Falls werden mikrobiologische Charakteristika des Isolats vorgestellt und diskutiert. Diese beinhalten phänotypische und genotypische Beschreibungen (auch unter Bezugnahme des gesamten bakteriellen Genoms).

## Introduction

*Photobacterium damselae* belongs to the family of the Vibrionaceae. The species was first described by Love et al. as *Vibrio damsela* in 1981 [1]. The first reclassification followed in 1985 and the species was included into the genus Listonella [2]. In 1991, Smith et al. undertook a reevaluation of the genus Listonella and *P. damselae* was finally introduced into bacterial taxonomy [3]. 

*P. damselae* has been detected in sea water and is a well-known fish pathogen [4], [5], [6]. The two haemoylsins damselysin (Dly) and phobalysin (PhlyP) have been identified as the main virulence factors for fish. The corresponding genes (the *dly* respectively *hlyA* gene) are encoded by the virulence plasmid pPHDD1 [5]. 

Besides this, infections in humans, occasionally with a fatal outcome, have also been described. In most of these cases, a previous contact with sea water or fish was reported and infections often originated from minor injuries, which many patients could not remember. Fatal courses of the disease were usually caused by a rapidly progressing necrotizing fasciitis, sepsis or are mediated by bacterial toxins. However, there are also localized infections of the skin, which mostly resulted in a complete healing.

Here we report on a persisting wound infection of a 32-year-old woman, which was caused by *P. damselae* after an accident in the Mediterranean. Furthermore, we also describe the bacterial isolate both phenotypically and genotypically.

## Case description

In August 2019, a 32-year-old female presented to our emergency department and reported that she had injured herself at a rotor leaf of a boat engine after falling off a dinghy into the salt water of the Mediterranean Sea on a trip to Spain 10 days before. Initially, the patient had received medical treatment by wound cleaning, disinfection and surgical stapling. Unfortunately, there is no information available on an antimicrobial therapy that has already been given in Spain.

Clinically we saw four laceration wounds (each approximately 4–5 cm) to the lateral thigh and calf of the left leg. While the two proximal wounds (thigh) were inconspicuous, the distal wounds on the calf displayed a local hyperemia, swelling and pressure pain. Additionally, there was slight bleeding and purulent secretion. The peripheral sensitivity, strength and mobility were unaffected. Moreover, the patient showed no systemic signs of infection. Except for a marginal elevation of the inflammation parameter CRP (14.7 mg/L) all further laboratory findings were normal. Radiographic imaging was not altered either. 

Apart from a nicotine (20 packyears) and alcohol abuse (2–3 drinks per day) the medical history of the patient was empty. 

The patient was admitted to the hospital and treated surgically. Intraoperatively, the swelling revealed to be an infected hematoma. After collection of microbiological samples, the hematoma was removed and the wound cavity was lavaged thoroughly. A drainage was inserted and the wound was then closed layer by layer. After the surgical treatment the patient received an immediate, empirical, intravenous antibiotic treatment with a cephalosporin (cefuroxime 4x 1.5 g per day). After confirmation of infection by *P. damselae*, the antibiotic treatment was adjusted to a combination of ampicillin (1 g) and sulbactam (2 g). The antibiotic was administered three times a day. After seven days of intravenous treatment, the patient was discharged from the hospital. We recommended an additional oral antibiotic treatment with amoxicillin (875 mg) and clavulanic acid (125 mg) for seven further days. The antibiotic was administered three times a day. A scheduled appointment for clinical reevaluation was not met by the patient. 

## Microbiological methods and results

### Cultivation

**Figure 1:**
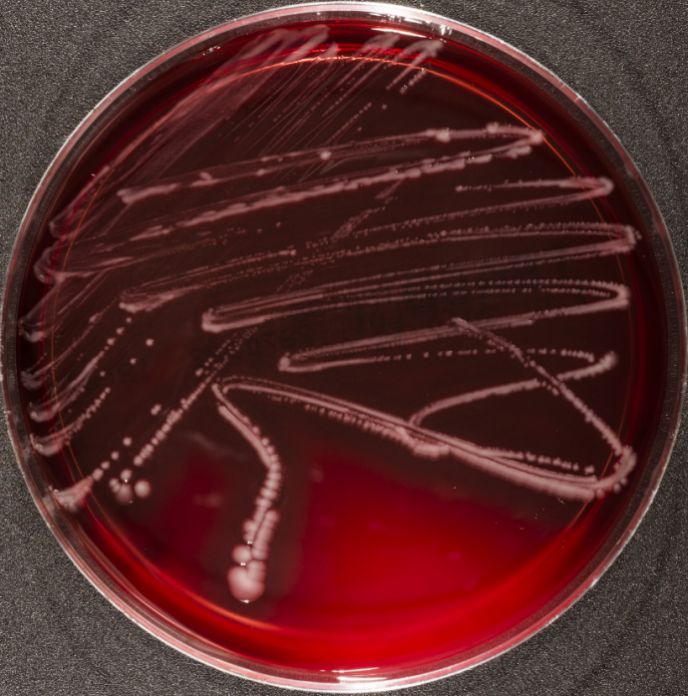
*Photobacterium dampselae* DSM 110634 growing on Columbia blood agar containing 5% sheep blood (Oxoid, Wesel, Germany). The bacteria were incubated for 18 hours.

In total, three specimens were sent to the Institute for Medical Microbiology and Hygiene of the Technical University Dresden: the first sample was collected from a wound swab (collected one day prior to the operation, isolate DSM 110633). The second (intraoperative wound swab, isolate DSM 110632) and the third sample (biopsy, isolate DSM 110634) were obtained in the course of the operation. The samples were cultured on Columbia agar with 5% sheep blood (Oxoid, Wesel, Germany), bile chrysoidin glycerol agar (Oxoid, Wesel, Germany), brain heart infusion (Becton Dickinson, Heidelberg, Germany) and Schaedler broth (bioMérieux, Nürtingen, Germany). The agar plates were incubated for 18 hours at 37ºC (without CO_2_ infusion). After incubation, smooth, glossy and slightly transparent bacterial colonies showing a strong beta haemolysis were detected on all Columbia blood agars (Figure 1 (Fig. 1)). Bacterial growth was also detected in brain heart infusion (indicated by turbidity) and bile chrysoidin glycerol agar.

### Identification 

MALDI-TOF MS (Bruker Daltonik, Bremen, Germany) was used for primary species identification. All isolates were identified as *P. damselae* with score values >2.0, which indicates a high confidence identification (DSM 110632: 2.131; DSM 110633: 2.334; DSM 110634: 2.395). The results were additionally confirmed by sequencing of the 16S rRNA gene using 27F (agagtttgatcmtggctcag) as forward and 1498R (cggttaccttgttacgactt) as reverse primer. The data were analysed using the BLAST algorithm (https://blast.ncbi.nlm.nih.gov/Blast.cgi). The species *P. damselae* was confirmed in all isolates (99% identity). The PCR product covers a length of 1,378 bases. A total of four mismatches were found.

### Antimicrobial susceptibility testing

Bacterial colonies originating from the third specimen (DSM 110634, biopsy) were inoculated in physiological sodium chloride solution (Fresenius, Bad Homburg, Germany) and a McFarland standard of 0.5 was created. The bacterial suspension was plated on Mueller-Hinton agar (bioMérieux, Nürtingen, Germany) using a plate rotator (bestbion dx, Köln, Germany). Gradient diffusion test strips (bestbion dx, Köln, Germany) were then placed on the agar plates and incubated at 37ºC for 18 hours. The interpretation of the MIC values was performed according to the EUCAST guidelines (PK/PD breakpoints) published in 2020 [7]. The isolate was susceptible towards ampicillin (0.25 mg/L), ampicillin sulbactam (0.25 mg/L), amoxicillin-clavulanate (0.5 mg/L), piperacillin (0.25 mg/L), piperacillin-tazobactam (0.064 mg/L), cefotaxime (≤0.016 mg/L), ceftazidime (0.125 mg/L), imipenem (0.5 mg/L), meropenem (0.032 mg/L), ciprofloxacin (0.008 mg/L), levofloxacin (0.004 mg/L) and moxifloxacin (0.016 mg/L). However, it showed resistance towards the aminogylcosides gentamicin (1.0 mg/L) and amikacin (4.0 mg/L). There are no breakpoints available for fosfomycin since there is insufficent evidence for its usefulness in the clinical setting. 

### Next generation sequencing and whole genome data analysis 

It could be assumed that all the isolates were identical since *P. damselae* is only extremely rarely detected as a pathogen in humans and beyond that, only pure cultures were found. For this reason, only one isolate (DSM 110634) was chosen for whole genome sequencing. Sequencing of a Nextera XT DNA Library (Nextera XT DNA Library Prep Kit; Illumina, San Diego, CA, USA) was performed on an Illumina NextSeq 550 instrument (Illumina, San Diego, CA, USA) followed by genome assembly using SPAdes 3.14 and an automated annotation applying NCBI Prokarytic Genome Annotation Pipeline [8], [9]. Screening for resistance genes was performed using ResFinder 2.1 as recently described and CARD5 [10], [11]. Pylogenomic analyses were carried out performing digital DNA-DNA hybridization (dDDH). Type (Strain) Genome Server (TYGS) was used as database [12]. The results showed an identity of 88.2% to *P. damselae* CIP 102761, thus confirming the species [13]. Resistance gene analysis using both ResFinder 2.1 and CARD 5 did not lead to an explanation for the observed resistance against aminoglycosides. The genome sequence was submitted to NCBI GenBank under accession number JAATTX000000000.

In total three genes encoding for haemoylsis were detected: *dly*, *hlyA* and (chromosomally encoded) *hylA*. Interestingly, the complete virulence plasmid pPHDD1 was not found. 

## Discussion

To the best of our knowledge, a total of 29 case descriptions of *P. damselae* infections in humans (including the present report) are described so far (Table 1 (Tab. 1)). All reports (except one) have in common that an infection has always been preceded by contact with sea water (the natural reservoir of *P. damselae*), fish or other sea animals (see Table 1 (Tab. 1)) [14]. Additionally, a closer look at the available literature reveals that fatal cases tend to affect (but are not exclusive to) older patients (n=10; average age=63.1 years). In contrast, there are young patients who have recovered from the infection (n=19; average age=38.9 years) (Table 1 (Tab. 1)). The patient in the present case was 32 years old. Based on our current knowledge, the course of the disease was also positive, which is congruent with the observations mentioned before. 

Basically, there are two possible explanations for a worse course of the infection. On the one hand, the deteriorating function of the immune system with age (immunosenescence) should be mentioned [15]. On the other hand, the different virulence properties of the strains must be taken into account. So far, the two haemolysins damselysin (Dly) and phobalysin (PhlyP) have been described as the most important virulence factors [5]. Our isolates also showed strong β-haemolysis. Genome analysis of the entire genome of the *P. damselae* strain DSM 110634 showed both haemolysins being present with 100% amino acid identity (Swissprot IDs D1J6Q4 and D1J6Q5). Both genes were found in one cluster similar to their assembly in pPHDD1. Moreover, the corresponding assembled contig, namely NODE_21, carried the *parA* replication gene showing clearly both haemolysin genes are encoded on a plasmid. However, the complete plasmid pPHDD1 (RefSeq ID NC_014653) was not found as such. Obviously, the virulence plasmid structure differs within our isolate. Surprisingly, a second chromosomally encoded haemolysin gene was found with 92% amino acid identity to *hlyA* (NODE_13:122725.120917). 

We were also able to reliably identify our isolate using the MALDI TOF MS since it was in accordance to results obtained from sequencing of the 16S rRNA gene and the dDDH. However, a general statement about the suitability of MALDI TOF MS for the identification of *P. damselae* can only be made using a larger collection of well-characterized strains derived from clinical specimens.

The data currently available are insufficient to make general statements about the antimicrobial resistance of *P. damselae*. However, previous reports suggest that the species is sensitive to most antibiotics (including most of the β-lactams, cotrimoxazole, aminoglycosides, fluoroquinolones) [16]. However, resistance to aminoglycosides (as in the present case report) has also been described [17]. Unfortunately, a search for genes providing aminoglycoside resistance using both ResFinder 2.1 and CARD 5 did not obtain any results. There are basically three possible explanations for the development of resistance to aminoglycosides:

1. reduction of the concentration of aminoglycosides within the bacterial cell (e.g. efflux pump),
2. changes in the target structure for aminoglycosides (e.g. 16S methylation or ribosomal mutations) and
3. enzymatic inactivation (e.g. aminoglycoside acetyltransferases, aminoglycoside nucleotidyltranserases, aminoglycoside phosphotransferases) [18].

It is important to note that amikacin is not inactivated by enzymes, which act on gentamycin or tobramycin [18]. However, both gentamycin and amikacin are resistant in our case. For this reason, a resistance mechanism is suggested in our isolate, which affects the entire class of aminoglycosides (e.g. an efflux pump). 

## Conclusion

We describe the 29^th^ case of a human infection caused by the marine bacterium *P. damselae*. Infections affecting younger patients (like the patient described here) seem to show a more favorable course of healing than those affecting older patients. Since the patient in this report did not keep a follow-up visit at the hospital after the therapy, we assume the healing process has been uncomplicated. Using whole genome sequencing, we could detect three haemolysins serving as virulence factors. However, the issue whether those proteins really contribute to human infections as well needs to be elucidated in more detail in further studies. Moreover, a larger strain collection is needed to be able to create a reliable resistance profile. 

## Notes

### Competing interests

The authors declare that they have no competing interests.

### Acknowledgements

The authors thank Franziska Klann and Stefan Tiede for excellent technical assistance and Thomas Riedel for support regarding strain deposition.

## Figures and Tables

**Table 1:**
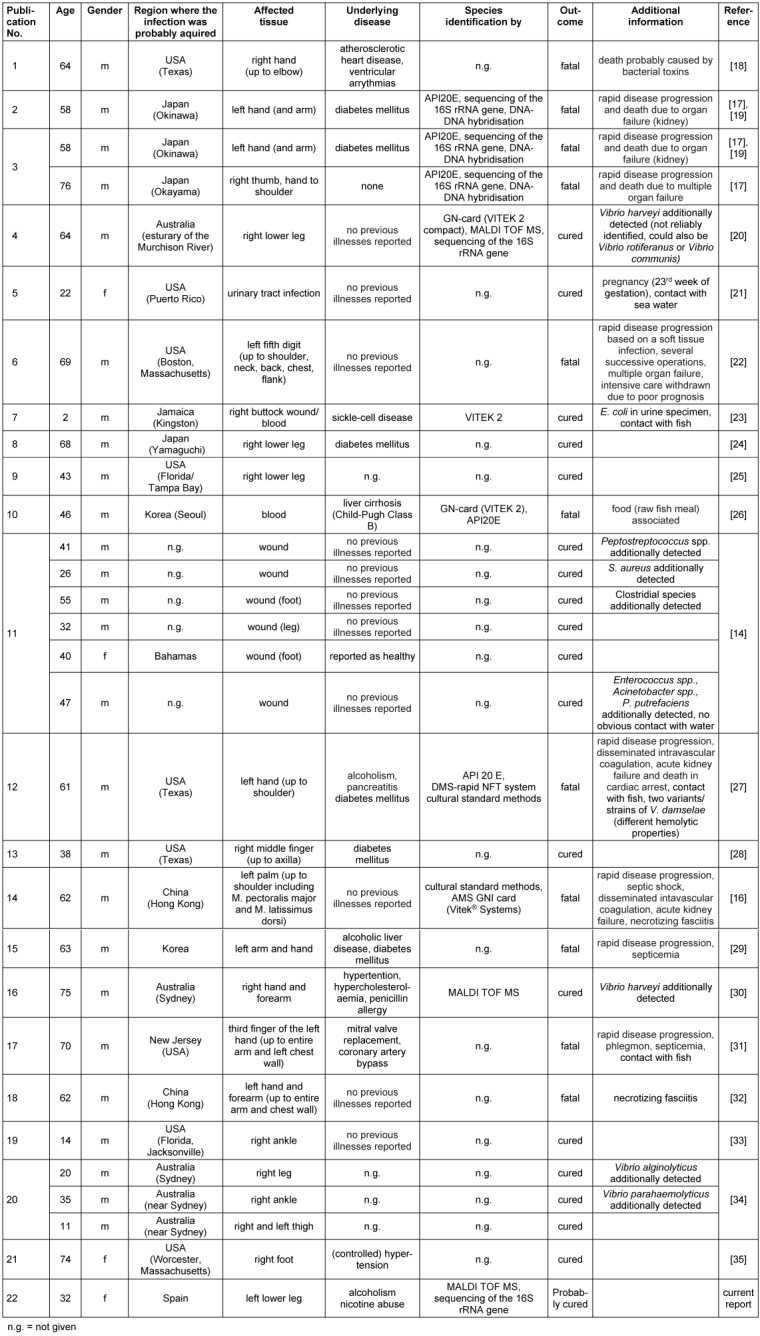
Overview of reports on human infections caused by *Photobacterium damselae*
